# Quantitative assessment of ohmic-type CdTe sensor response in a photon-counting X-ray imaging detector under continuous 12–49 keV irradiations

**DOI:** 10.1107/S1600577525004576

**Published:** 2025-06-26

**Authors:** Fabienne Orsini, Yasuhiko Imai, Takaki Hatsui

**Affiliations:** a RIKEN SPring-8 Center, 1-1-1 Kouto, Sayo-cho, Sayo-gun, Hyogo679-5148, Japan; b Japan Synchrotron Radiation Research Institute 1-1-1 Kouto Sayo Hyôgo679-5198 Japan; University of Malaga, Spain

**Keywords:** cadmium telluride sensors, hybrid pixels, X-ray irradiation, synchrotron radiation experiments, absorbed doses, polarization effects

## Abstract

For several years, photon-counting X-ray imaging detectors with cadmium telluride (CdTe) sensors have been used in high-energy synchrotron experiments. While these detectors exhibit excellent detection sensitivity at high energy, concerns remain regarding their performance stability over time under exposure to high-energy X-rays, an issue that can be critical for certain experiments. This study aims to quantitatively assess the response of ohmic-type CdTe sensors under well defined conditions of continuous X-ray irradiation, considering dose rate, photon energy and average absorbed dose throughout the sensor depth. Measurements were performed in a laboratory environment using a dedicated setup with a reliable and reproducible measurement protocol. The results revealed significant irradiation-induced performance variations over time. Notably, a loss of more than 11% in photon counts was observed, even at a relatively low photon flux of 5000 photons s^−1^ pixel^−1^ at 49 keV. The key contribution of this work is a quantitative characterization of the behavior of CdTe sensors within the 12–49 keV energy range under controlled conditions. These findings provide essential insights for synchrotron experiments operating in this energy range. Furthermore, the proposed measurement protocol offers a reliable method for quantitatively comparing the stability of other high-*Z* sensor materials against state-of-the-art CdTe technology.

## Abstract 2

This paper presents the evaluation results of the response of a pixelated photon-counting detector, equipped with ohmic-type cadmium telluride sensors, under high-energy X-ray irradiations.

## 1. Introduction

SPring-8-II is a major upgrade project to SPring-8, which was inaugurated in October 1997 as a third-generation synchrotron radiation light source. SPring-8-II will achieve a significantly lower electron emittance of 50 pm rad while substantially reducing power consumption (Tanaka *et al.*, 2024 ▸). The lower emittance by 48× will result in a more than two-orders-of-magnitude increase in brilliance within the 35–80 keV photon energy range.

From the perspective of X-ray imaging detectors, achieving both high quantum efficiency and stability, under continuous X-ray irradiation in this photon energy range, presents a significant challenge. In particular, maintaining detector response stability over time is a critical factor, as it directly impacts data quality in various synchrotron experiments, such as diffraction of single crystals or total scattering of various materials, where pair distribution function analysis is performed.

CdTe sensors have become widely used at SPring-8 for photon energies above 30 keV and, for now and in the coming years, remain the only material used in large-area detectors (greater than 10 cm × 10 cm) and suitable for high-energy synchrotron applications. Despite the long history of CdTe sensor development, these detectors still exhibit internal spatial defects and performance instabilities over time under irradiation. Unfortunately, these effects have not been sufficiently quantified in the literature under synchrotron radiation conditions for photon energies above 30 keV. This time-dependent performance degradation, commonly referred to as ‘polarization’ (Toyama *et al.*, 2006 ▸), is an instability observed in semiconductor detectors, leading to a gradual decline in performance, including photon count loss and reduced charge collection efficiency.

The ‘polarization’ effect can be observed after applying the bias voltage and has been widely reported in CdTe sensors with asymmetric Schottky contacts (Astromskas *et al.*, 2016 ▸; Meyer *et al.*, 2022 ▸) and symmetric ohmic contacts (Ruat & Ponchut, 2014 ▸; Pennicard *et al.*, 2014 ▸). In the case of Schottky contacts, polarization effects are quite severe and typically require periodic bias voltage cycling or temperature annealing. Although less severe, this effect is still present in ohmic-contact sensors, especially under high X-ray fluxes and prolonged irradiation, even after one or several hours of bias voltage stabilization. From a data quality perspective, any changes in detector response following polarization effects are critically important, and these effects have yet to be quantitatively assessed.

In this study, we present a measurement protocol designed to evaluate the ‘polarization’ effect after the stabilization of bias-induced polarization, under conditions that simulate synchrotron experiments. This methodology is based on laboratory X-ray sources, offering several advantages, including the elimination of beam time constraints, which enables systematic studies. Notably, this protocol is also accessible for detector manufacturers to implement. However, it is important to acknowledge that the available photon energy range is limited. In this study, measurements were conducted within the 12–49 keV energy range.

Section 2 details the dedicated laboratory setup and measurement protocol, followed by Section 3, which presents the results of X-ray irradiation on CdTe sensors across various photon energies and dose rates. These results provide quantitative insights into irradiation-induced variations in photon-counting performance, including the rate of change, the magnitude of the effect, and the conditions under which these variations occur in terms of photon flux and energy. Finally, Section 4 discusses the implications of these findings for synchrotron radiation experiments at SPring-8.

## 2. Equipment and protocol applied

### 2.1. CdTe detector and X-ray source

The CdTe detector used for the irradiation tests is a photon-counting LAMBDA 750k detector (X-Spectrum, 2022 ▸). It features CdTe sensors manufactured by Acrorad (Funaki *et al.*, 2007 ▸), with a pixel pitch of 55 µm and a thickness of 1 mm. The CdTe sensors, of *p*-bulk type, use ohmic contacts, and the default applied bias voltage is −300 V on the front side of the sensor, leading to electron collection on the back side with pixel contacts. The readout chip of this detector is MEDIPIX3 (Ballabriga *et al.*, 2007 ▸), with settings of the front-end chips optimized for the detection of high-energy photons, and the CdTe sensors feature a leakage current in the 20 nA range per sensor. The detector is used in a conservative 24-bit mode, with a shutter time of 10 s per image and a low frame rate of ≤1 Hz. In this mode, a single energy threshold is used and applied to all pixels, set to half the energy of incoming photons to minimize the charge-sharing effect. The charge summing mode is disabled. As recommended in the detector’s user manual, the water chiller temperature was maintained at 20°C throughout all measurements, resulting in a stabilized sensor temperature of approximately 35°C.

The X-ray tube used in this study is a COMET MXR-160 model (https://xray.comet.tech/en/products/mxr-160-22) with a tungsten anode. It operates at voltages of up to 160 kV and a maximum current of 18.75 mA. The long-term stability of the X-ray source has been demonstrated to be within ±0.1% of the set current (in milliamps) after a 1 h warm-up and for durations exceeding 8 h (COMET power supply datasheet, Ivario-160/4.5). Both the X-ray source and the detector are remotely controlled.

### 2.2. Laboratory setup for irradiation tests

**Figure 1:**
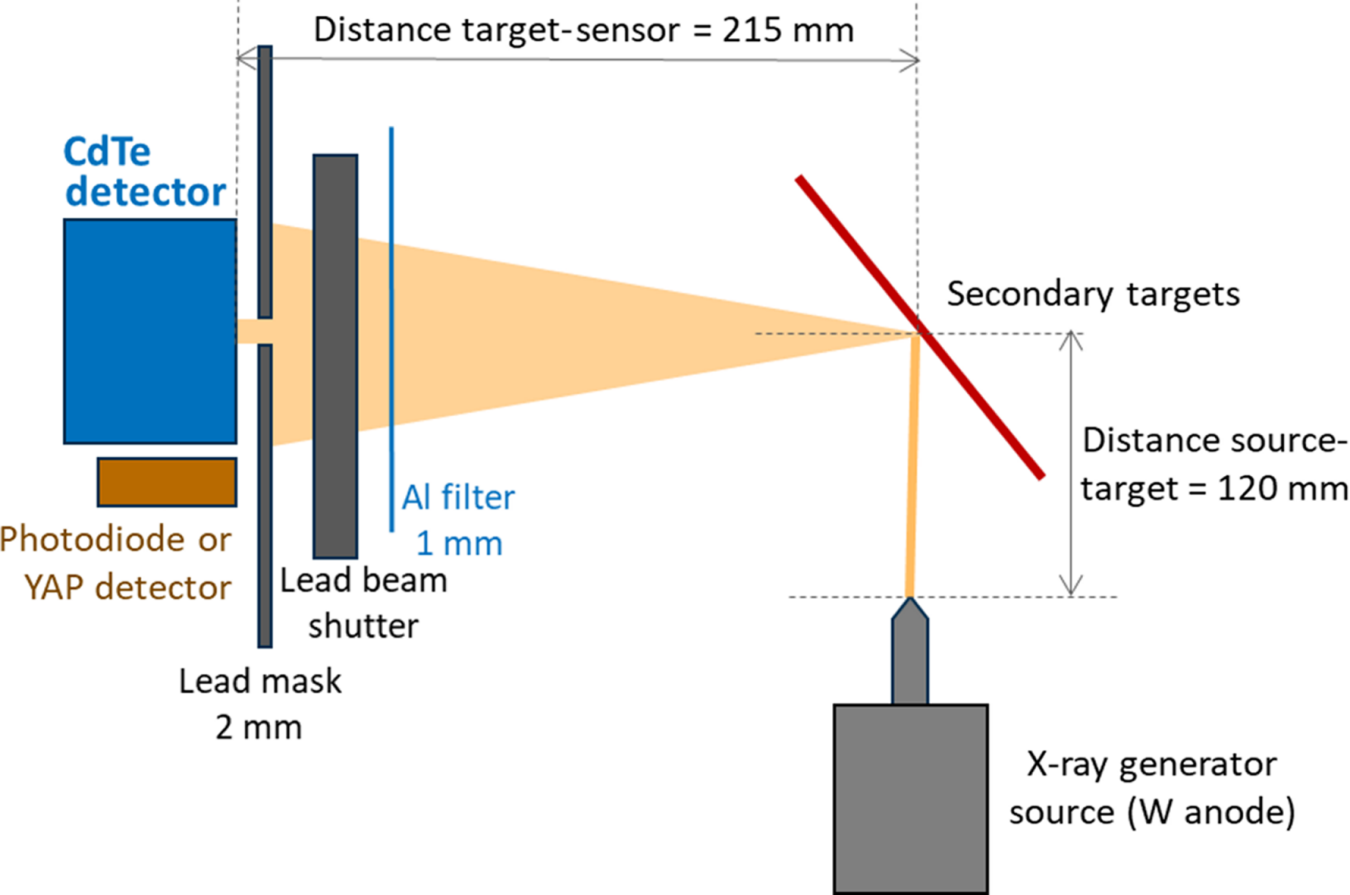
Setup used for evaluating CdTe sensor performance under X-ray irradiation. The secondary targets allow the selection of different elemental materials. A 1 mm-thick aluminium filter removes *L* lines from high-*Z* elements, whereas a 2 mm-thick lead mask ensures that only the region of interest (RoI) in the CdTe sensor is irradiated with a perpendicularly incident beam.

The experimental setup is illustrated in Fig. 1 ▸. In our measurements, the X-ray source irradiates secondary targets, generating fluorescence radiation from the *K* lines of the following elements: Ba (11.9 keV), Br (32.2 keV), Eu (41.5 keV) and Er (49.1 keV). The energy spectrum of each target was verified prior to the measurements using an energy-dispersive detector (CdTe Solid State Detector). To optimize the X-ray beam, a 1 mm-thick aluminium filter is used to eliminate *L* lines from high-*Z* elements. Additionally, a 2 mm-thick lead mask, positioned close to the sensor, ensures that only the central portion of the photon beam is selected, defining a region of interest (RoI) while avoiding unnecessary irradiation of the entire detector. A thick lead beam shutter, mounted on a motorized stage, is integrated into the setup to block X-ray irradiation when required, as the X-ray source itself lacks an output shutter. The CdTe detector is mounted on motorized horizontal and vertical stages, enabling precise positioning and repositioning of specific areas of interest.

In the following, the photon flux has been measured by both the photon-counting detector and an Si PIN photodiode (S-2500, Ohyo Koken Kyogo Co. Ltd) or a YAP(Ce) detector (HX-101, Ohyo Koken Kyogo Co. Ltd). Due to the spectral profile of our X-ray source, which decreases at higher energies, the photon flux reaching the sensor from the *K* line emissions of the secondary targets is less intense. As a result, the irradiation duration – a key parameter in determining the absorbed dose – is adjusted based on the working energy. Typically, short irradiation times of a few hours are used at 12 keV, whereas longer durations exceeding 15 h are required at 49 keV, depending on the total absorbed dose desired.

### 2.3. Timing sequence and measurement protocol

**Figure 2:**
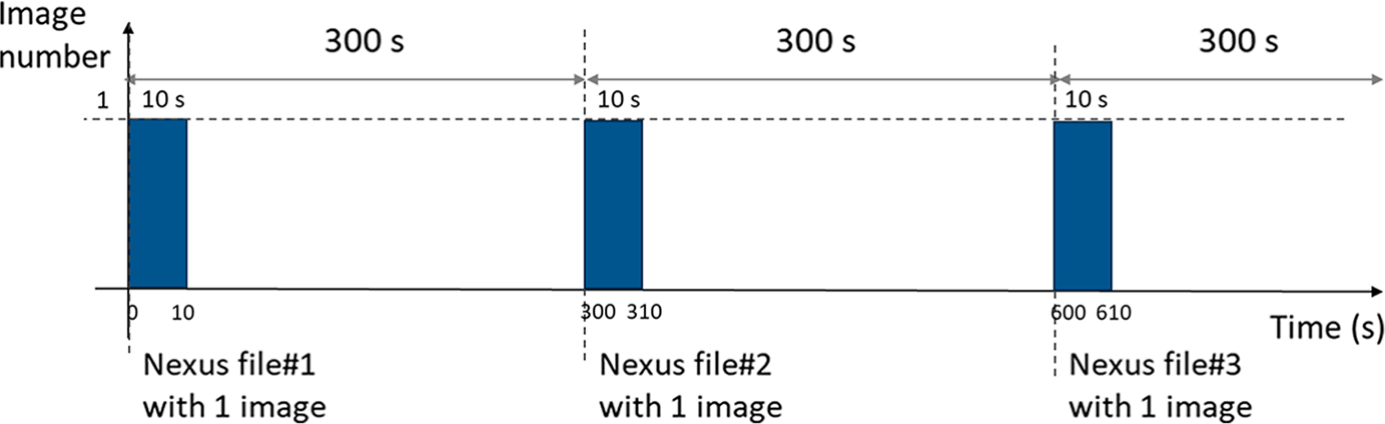
Timing sequence applied for the data acquisition and readout of the detector during continuous X-ray irradiations.

The timing sequence for data acquisition, illustrated in Fig. 2 ▸, is straightforward. The CdTe sensor is continuously irradiated with photons from the secondary target, and a single 10 s exposure is recorded every 300 s using the internal software trigger function. This approach ensures that each image contains a sufficient number of photon counts (more than several thousand, regardless of energy) to produce a Gaussian distribution with robust statistical significance. Given that individual measurements can last from several hours to tens of hours, the interval between consecutive images is optimized to provide an adequate number of data points without becoming excessive. Preliminary tests were conducted with different interval times and shorter acquisition durations to validate the method, confirming that the chosen timing sequence does not introduce any bias.

**Figure 3:**
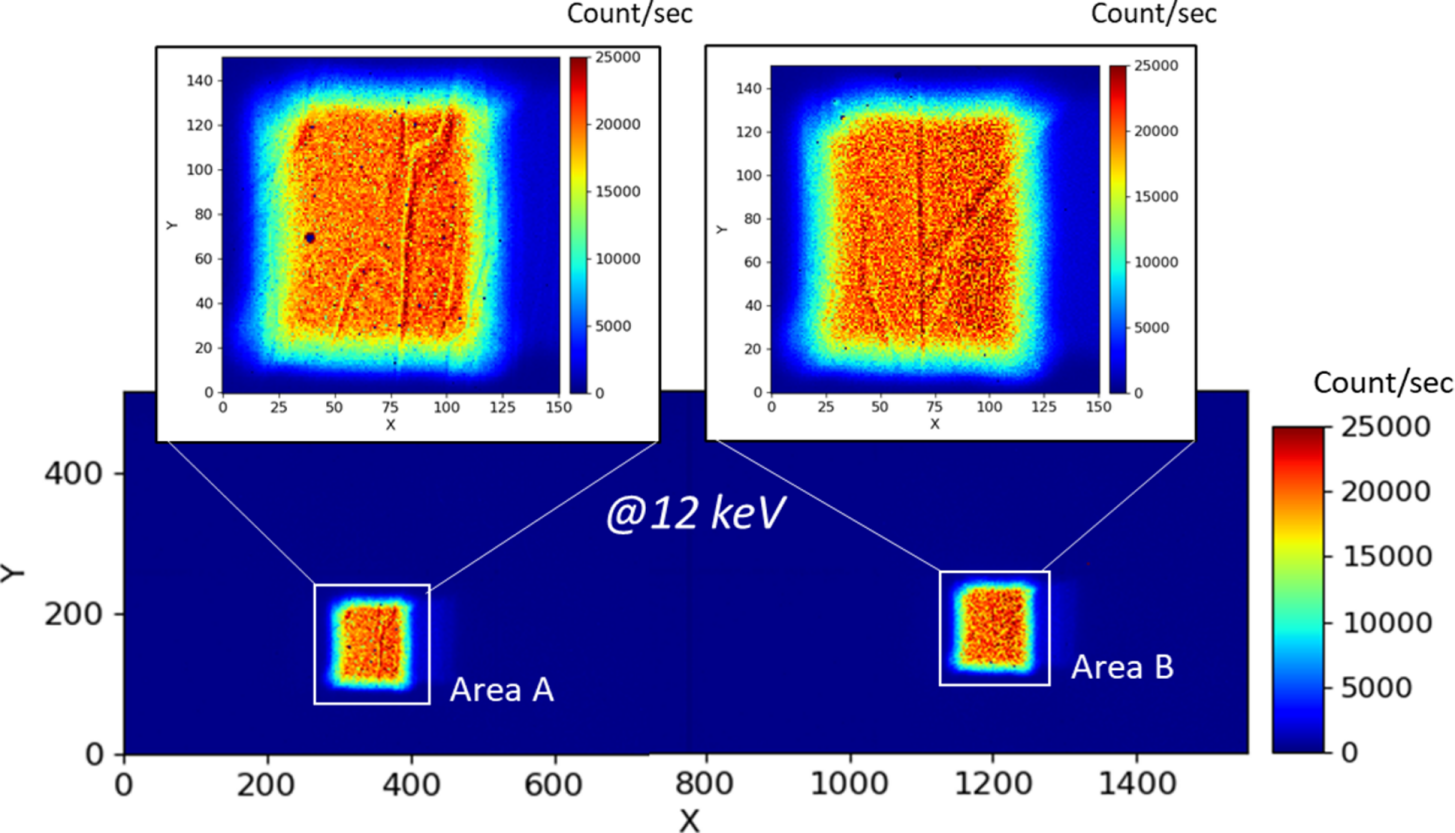
For all tested energies, two different areas of approximately 5.5 mm × 5.5 mm of the CdTe sensors are irradiated (one after the other). This example illustrates irradiation with 12 keV photons, resulting in a maximum photon count of approximately 20000 counts s^−1^ pixel^−1^.

As mentioned previously, four photon energies are used for the evaluation of the CdTe sensors: 11.9 keV, 32.2 keV, 41.5 keV and 49.1 keV. For each energy, two different areas labeled A and B are selected on the CdTe sensors by positioning the detector in front of the hole in the lead mask. The irradiated areas are approximately 5.5 mm × 5.5 mm (∼100 × 100 pixels) and are located far from the CdTe sensor edges, preventing any eventual different behaviors from the edge pixels. These two areas are intentionally chosen to include uniform regions, defect lines, and, in the case of area A, a ‘dead’ spot. An example showing the full detector surface with the selected working areas is presented in Fig. 3 ▸, where the photon energy is 12 keV.

For this study, calculating the absorbed dose is crucial for understanding the detector response to a specific quantitative irradiation. The dose quantifies the energy deposited per unit mass of the material absorbing the radiation. It is usually calculated using the formula *D* = 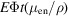 (Ravotti, 2018 ▸), where *D* is the dose (in Gy), *E* is the energy of the X-rays (in J), Φ is the photon flux (in photons s^−1^ m^−2^), *t* is the exposure time (in s), μ_en_/ρ is the mass energy absorption coefficient for CdTe (in m^2^ kg^−1^) and ρ is the density of CdTe material in kg m^−3^. The mass energy absorption coefficient μ_en_/ρ depends on the material through which the X-rays pass and the energy of the incoming X-rays. Values of μ_en_/ρ are taken from the NIST website (Hubbell & Seltzer, 2004 ▸). The peak dose at the surface can be calculated using the incoming photon flux. Nevertheless, to determine the average dose within the material of thickness *d*, while accounting for the absorption depth and the decrease in photon flux as they penetrate deeper, we need to integrate over the sensor depth as follows,
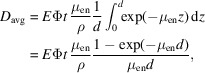
where *d* is the thickness of our material (= 1 mm in our case). In our energy range, 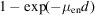 can be approximated to 1, leading to an average dose directly proportional to the photon energy.

At low energy photons like 12 keV, almost all photons deposit their energy within a thin layer of CdTe, leading to a high dose in a small volume, whereas, at 49 keV, photons distribute their energy over a greater depth, leading to a smaller dose at the surface, but the average dose is quite high as illustrated in Table 1 ▸. This table summarizes the values used in our measurements, with the relationship between the photon flux, the dose rate, the average dose throughout the pixel and the peak dose at the surface, at several photon energies. As shown in this table, the photon flux is on the order of 10^6^ photons s^−1^ mm^−2^ (<10000 photons s^−1^ pixel^−1^ for energies above 32 keV), which is representative of a ‘moderate’ flux during an experiment, ensuring that the detector remains in its linear counting region. In the following sections, the results are presented as a function of the average dose to better account for the dose deposited along the path within the pixel.

**Figure 4:**
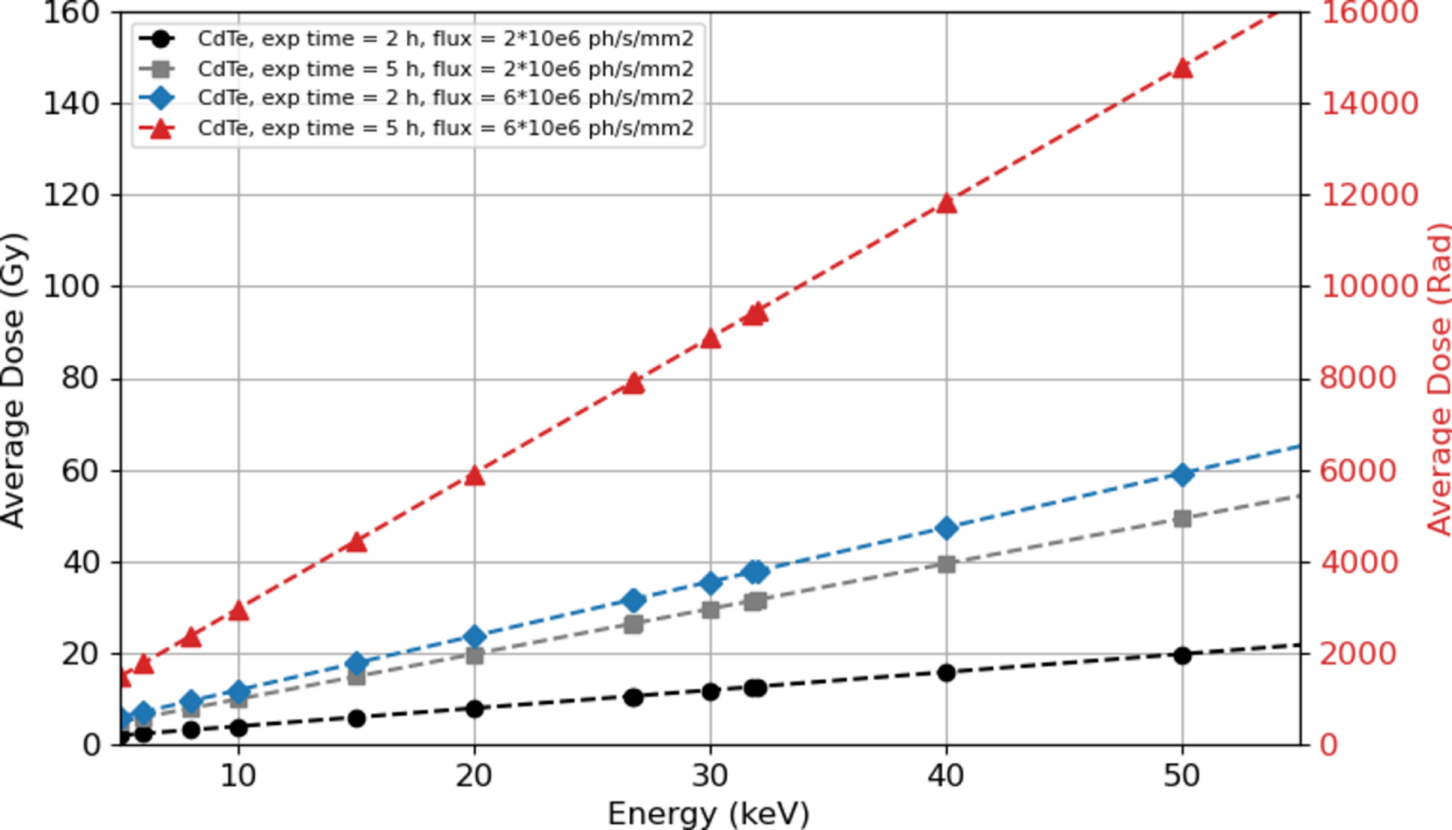
Average dose (in Gy) in a 1 mm-thick CdTe sensor as a function of photon energy. Several curves are provided as examples to illustrate photon fluxes accessible with our X-ray source.

Fig. 4 ▸ illustrates the average dose in 1 mm of CdTe sensor for different photon fluxes across the energy range from 5 keV to 55 keV, which are typical of ‘moderate’ irradiation during a synchrotron experiment. For example, at energies below 20 keV, with a ‘moderate’ flux of 6 × 10^6^ photons s^−1^ mm^−2^ and an irradiation duration of about 5 h (as shown by the red curve), the sensor is not expected to absorb more than 60 Gy.

The protocol applied to the detector is divided into two parts. The first part involves positioning the CdTe sensor precisely in the desired location (area A or B) and checking the initial photon count (see Table 1 ▸). For this step, the CdTe sensor is irradiated with the selected energy for a very short duration (1 to 10 s maximum). Once the position is confirmed and the photon flux is adjusted to the desired value, the sensor bias voltage is set to 0 V for at least 1 h. The second part is the long-duration irradiation measurement. During this phase, the sensor is initialized without X-rays (the X-ray source is powered on for the warm-up phase, and the beam shutter is placed in front of the sensor). The bias voltage is checked to ensure it is stabilized and is continuously monitored throughout the measurement. Before starting irradiation, the detector was kept under bias voltage for 1 h without exposure. This waiting period was chosen based on previous low-irradiation measurements, which showed that the detector response stabilizes after 50 min to 1 h. Before each irradiation measurement, a dark image is always captured to check the detector’s state. At the end of each irradiation measurement, the whole detector is powered off. A minimum of 3 h, up to an entire night, must pass before starting a new measurement. These two parts of the protocol are applied to each measurement, making the test campaign lengthy but highly systematic, yielding precise results (the repeatability of the measurements has been cross-checked).

## 3. Assessment of the stability of the CdTe sensor response under X-ray irradiation

Once a selected area has been irradiated, the data analysis process remains consistent for all measurements. A sub-matrix of 75 × 75 pixels is chosen within areas A or B to avoid edge effects caused by the lead mask. The first image, representing the initial state, is used to normalize all subsequent images from the same measurement. The number of counts in a homogeneous area (Section 3.1), along a defect line (Section 3.2.1) and around a ‘dead’ spot (Section 3.2.2) inside this sub-matrix is tracked over time. The evolution of counts as a function of the average dose is then plotted graphically. Note that all curves presented are normalized to the initial image taken at the beginning of the irradiation.

### 3.1. Irradiation effect on a homogeneous area

**Figure 5:**
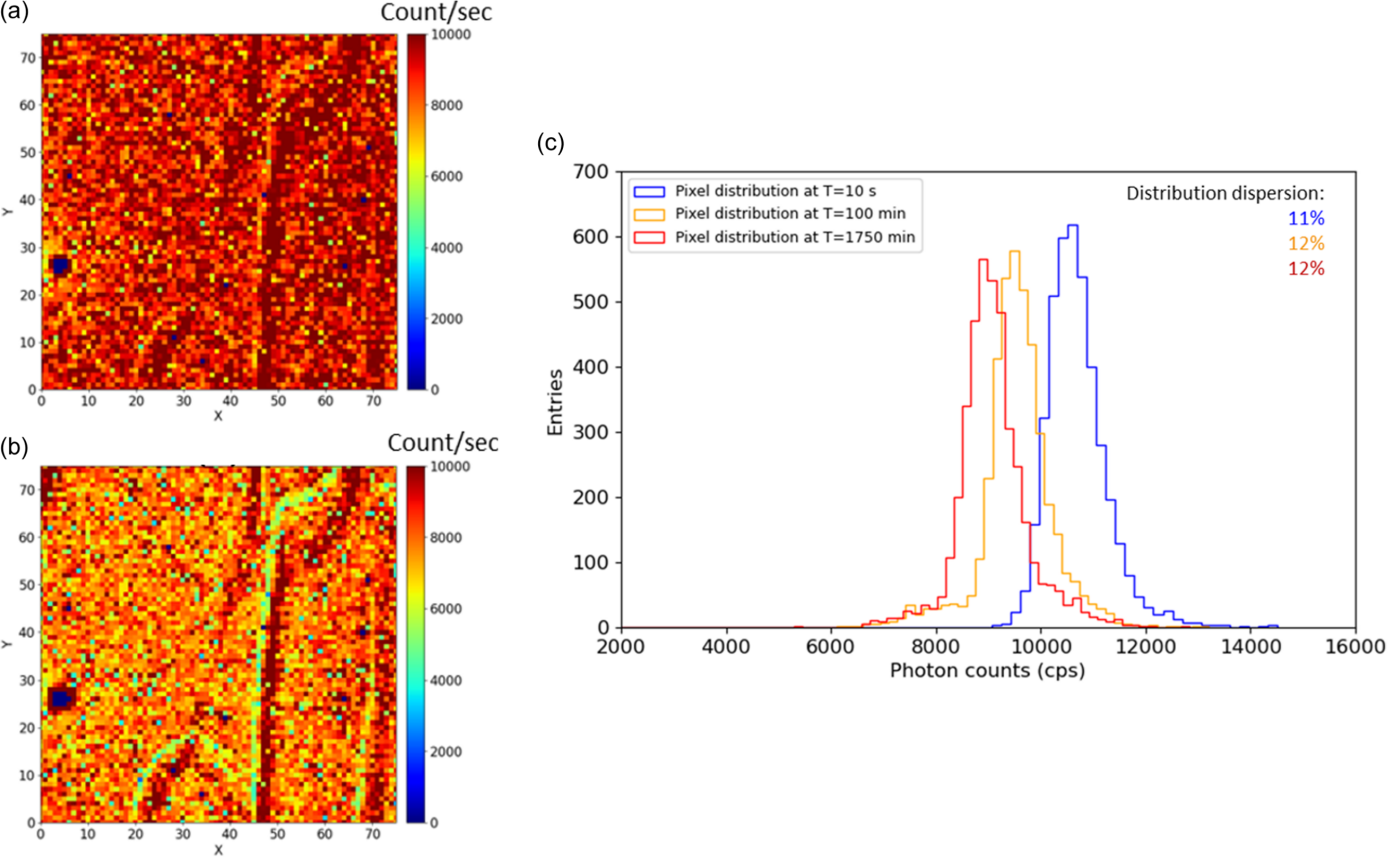
Example of the CdTe sensor response under irradiation at 41 keV in area A: (*a*) initial hit map at the beginning of irradiation and (*b*) at the end of irradiation for an average dose of 389 Gy. (*c*) Histograms of count distribution at three different times during irradiation. The sigma values of the distributions are in the range 11% to 12%.

**Figure 6:**
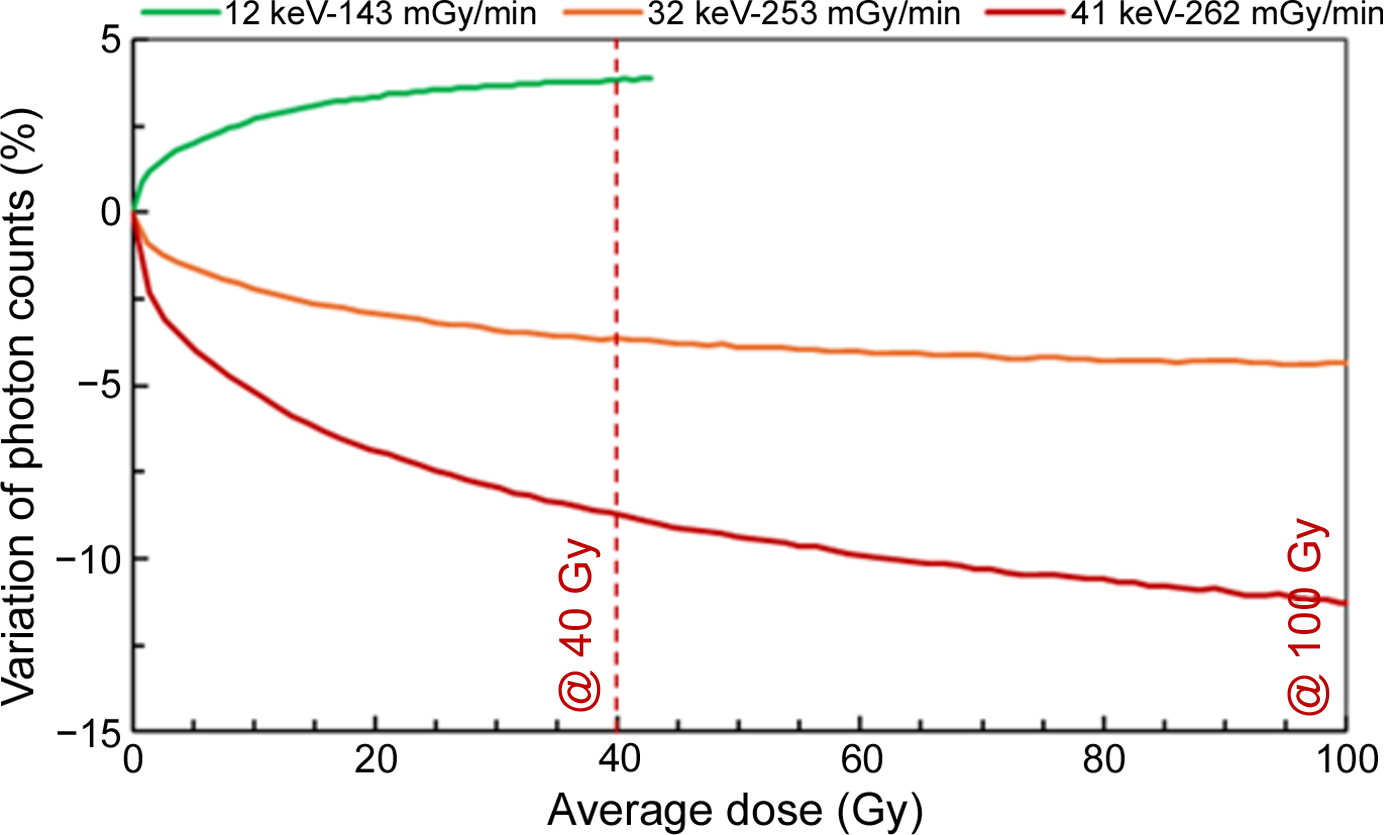
Variation of photon counts in area B as a function of average dose, for different energies and corresponding dose rates.

Fig. 5 ▸ illustrates the irradiation effect within area A at 41 keV. The hit map of the sub-matrix at the beginning of the irradiation is shown in Fig. 5 ▸(*a*), and Fig. 5 ▸(*b*) depicts the same area after irradiation equivalent to 389 Gy at an average dose rate of 222 mGy min^−1^. The histogram of photon count distribution before and after irradiation is shown in Fig. 5 ▸(*c*). The sigma values of these distributions range from 11% to 12% and remain unchanged by irradiation. The loss of counts over time is clearly visible, with a shift in the distribution toward lower counts. The total loss of counts in area A is −14% after 389 Gy at an average dose rate of 222 mGy min^−1^ at 41 keV. The most significant loss occurs in the first 30 Gy to 40 Gy (depending on the photon energy), after which the count loss slows down and gradually stabilizes. This behavior is further illustrated in Figs. 6 ▸ and 7 ▸ and discussed in Section 4.

Fig. 6 ▸ illustrates the variation of counts versus the average dose in area B for several associated dose rates and energies. Two key conclusions can be drawn from this figure. First, regardless of the dose rate, a loss of photon counts is observed, particularly within the first 30 Gy to 40 Gy, for all energies. Reaching an average dose of 100 Gy or more takes a long time (over 30 h at low dose rates for high-energy photons). Since the CdTe sensor tends to reach a ‘plateau’ after approximately a few dozen Gy, subsequent measurements were limited to low dose values. The second conclusion is the clear dependence of the loss of counts on X-ray energy, particularly when the energy exceeds 12 keV. As the following results confirm, higher energy leads to a greater variation of the detector response over time. In this figure, at 12 keV, irradiation was halted after 40 Gy due to the high dose peak at the surface, as the ‘plateau’ region had already been reached. At this photon energy, the interactions occur near the front side surface. The internal electric field is consequently not or less affected by the polarization effect, leading to an efficient collection of charges, appearing as a slight enhancement in sensitivity of 3% to 4%.

**Figure 7:**
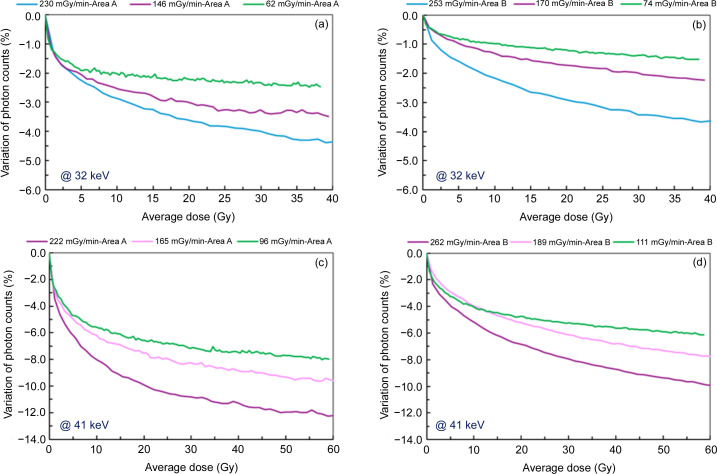
Variation of photon counts as a function of the average dose for several dose rates: at 32 keV in areas (*a*) A and (*b*) B, and at 41 keV in areas (*c*) A and (*d*) B.

To more precisely quantify the correlation between the variation in counts with dose rate and energy, the following measurements were performed: the energy of the incoming photons was fixed and the photon flux was varied within the capabilities of our X-ray source. Fig. 7 ▸ presents the results obtained at 32 keV in area A [Fig. 7 ▸(*a*)], in area B [Fig. 7 ▸(*b*)] and at 41 keV in area A [Fig. 7 ▸(*c*)], in area B [Fig. 7 ▸(*d*)]. For a fixed photon energy of 32 keV, three different dose rates were applied: 230 mGy min^−1^, 146 mGy min^−1^ and 62 mGy min^−1^ in area A [Fig. 7 ▸(*a*)]. A correlation between the loss of counts and higher dose rates is observed in both areas A and B [Figs. 7 ▸(*a*) and 7 ▸(*b*)]. The same behavior is observed at 41 keV [Figs 7 ▸(*c*) and 7 ▸(*d*)], but the variation in photon counts, at an average dose equivalent of 40 Gy, is more pronounced at 41 keV compared with 32 keV. For example, in Area A, at a dose rate of 220 to 230 mGy min^−1^, the photon count loss after 40 Gy of irradiation increases from −4.3% at 32 keV [blue curve in Fig. 7 ▸(*a*)] to −11.3% at 41 keV [purple curve in Fig. 7 ▸(*c*)]. A similar trend is observed in Area B, at a dose rate of 250 to 260 mGy min^−1^, where the photon count loss after 40 Gy of irradiation reaches −3.6% at 32 keV [blue curve in Fig. 7 ▸(*b*)] and −8.7% at 41 keV [purple curve in Fig. 7 ▸(*d*)].

**Figure 8:**
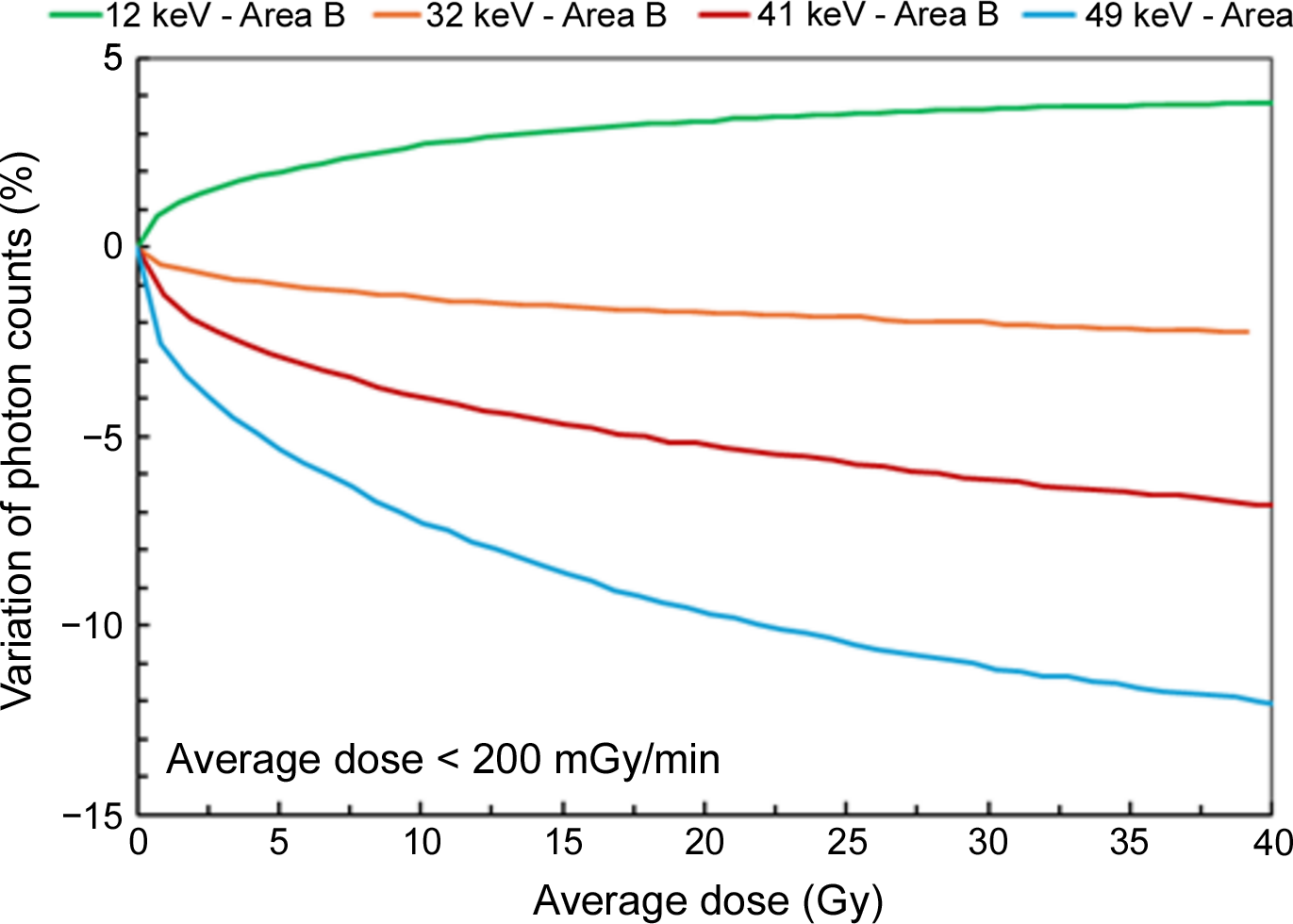
Variation of photon counts in area B as a function of the average dose for several photon energies, at a dose rate of <200 mGy min^−1^.

Based on previous results, the photon energy during irradiation appears to have a significant effect on the variation in the CdTe sensor’s response. Fig. 8 ▸ illustrates the effect of photon energy on the observed instability in area B (a similar trend is seen in area A). In this case, the dose rate is fixed at a value below 200 mGy min^−1^. A correlation with the incident photon energy is observed. For instance, in Fig. 8 ▸, the loss of counts after 40 Gy irradiation varies from +3.8% at 12 keV to −2.3% at 32 keV, to −6.8% at 41 keV and to −12.1% at 49 keV. The variation in the detector response is not negligible at energies equal to or above 32 keV.

**Figure 9:**
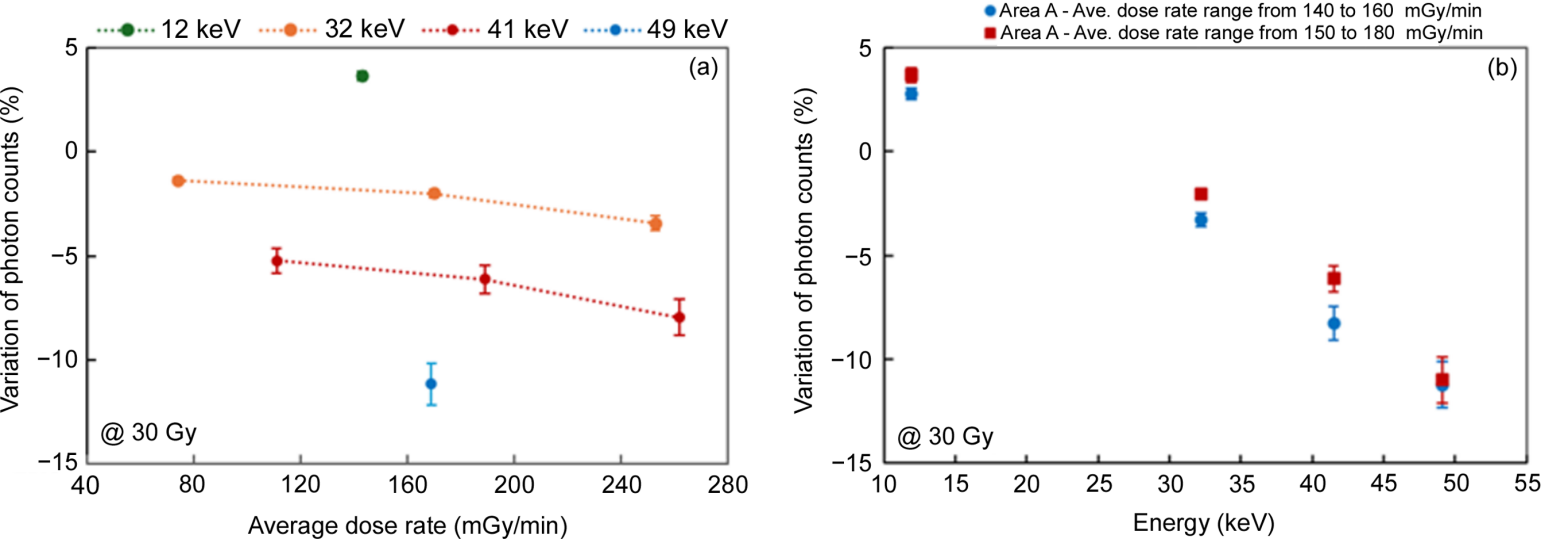
Variation in photon count after irradiation equivalent to 30 Gy: (*a*) as a function of the average dose rate at various energies in Area B; and (*b*) as a function of energy in Area A for a dose rate of 140–160 mGy min^−1^ and in Area B for a dose rate of 150–180 mGy min^−1^.

Fig. 9 ▸(*a*) highlights the influence of the dose rate on the degradation of performance due to irradiation at several photon energies. The higher the dose rate is, the greater the count loss. Fig. 9 ▸(*b*) summarizes the irradiation effect in both areas A and B for dose rates that are closely matched (140–160 mGy min^−1^ for area A and 150–180 mGy min^−1^ for area B). While the sensitivity to irradiation in the two areas is not strictly identical, the trend with photon energy dependence is very similar.

A possible explanation for the mechanism occurring in the sensor is discussed in Section 4, along with the implications for high-energy synchrotron experiments.

### 3.2. Effect of irradiation on defective areas

#### 3.2.1. Irradiation effects on pixels in defect lines

**Figure 10:**
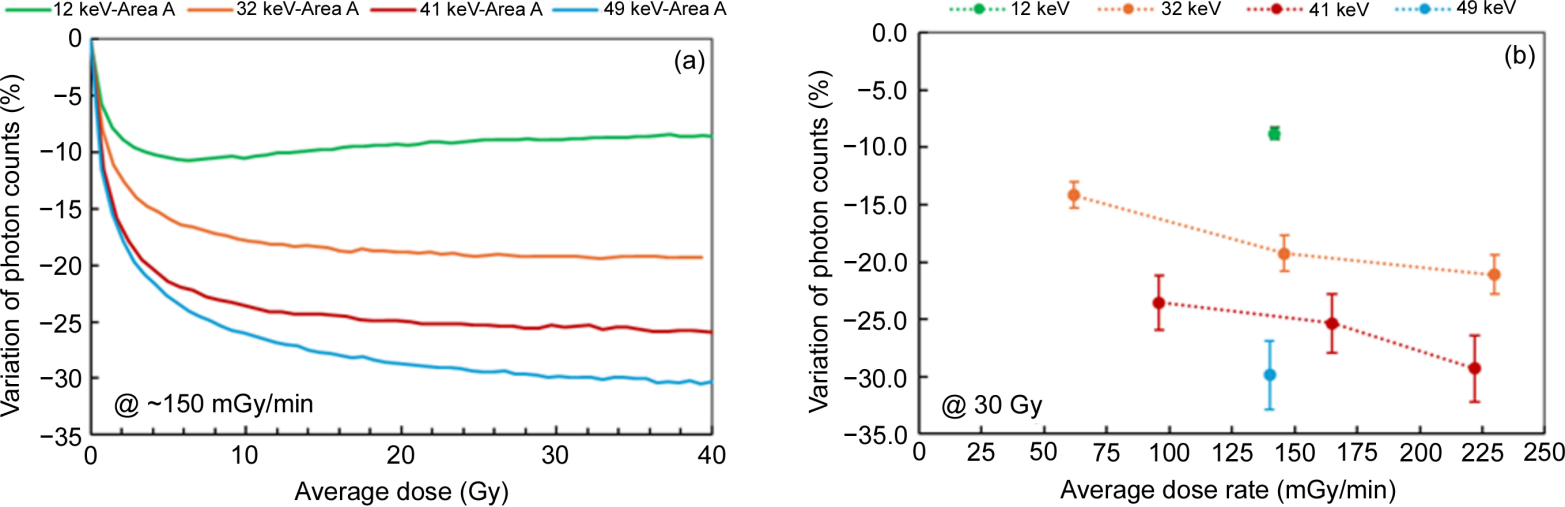
Variation of photon counts along the defect line in area A: (*a*) as a function of the average dose for several photon energies at a dose rate of ∼150 mGy min^−1^; and (*b*) as a function of the dose rate, for an average dose of 30 Gy, for several photon energies.

In the previous section, the results were presented by tracking pixels within a ‘uniform’ sub-matrix in areas A or B, excluding pixels located on defect lines or near them. Specific data analysis was conducted by focusing solely on pixels from a ‘low-intensity’ defect line in both areas, where the initial photon count is lower compared with other pixels. The results of the defect line pixel response in area A are presented in Fig. 10 ▸.

The same effect of count loss is observed for these specific pixels, but the level of variation is much greater than for those in a ‘uniform’ area. For example, at a dose rate of about 150 mGy min^−1^, the variation in counts at the end of the 30 Gy irradiation ranges from −8.8% at 12 keV, −19.2% at 32 keV, to −25.4% at 41 keV and −29.8% at 49 keV. These levels of variation are significant and are not corrected by the flat-field image (which is static and taken before the experiment). Since the detector likely contains many defective lines, as is common with CdTe sensors, it is recommended to conduct experiments using sample signals outside these areas. However, this is not always feasible, particularly in scattering experiments with rings or elongated patterns.

#### 3.2.2. Irradiation effects on pixels close to dead spots

**Figure 11:**
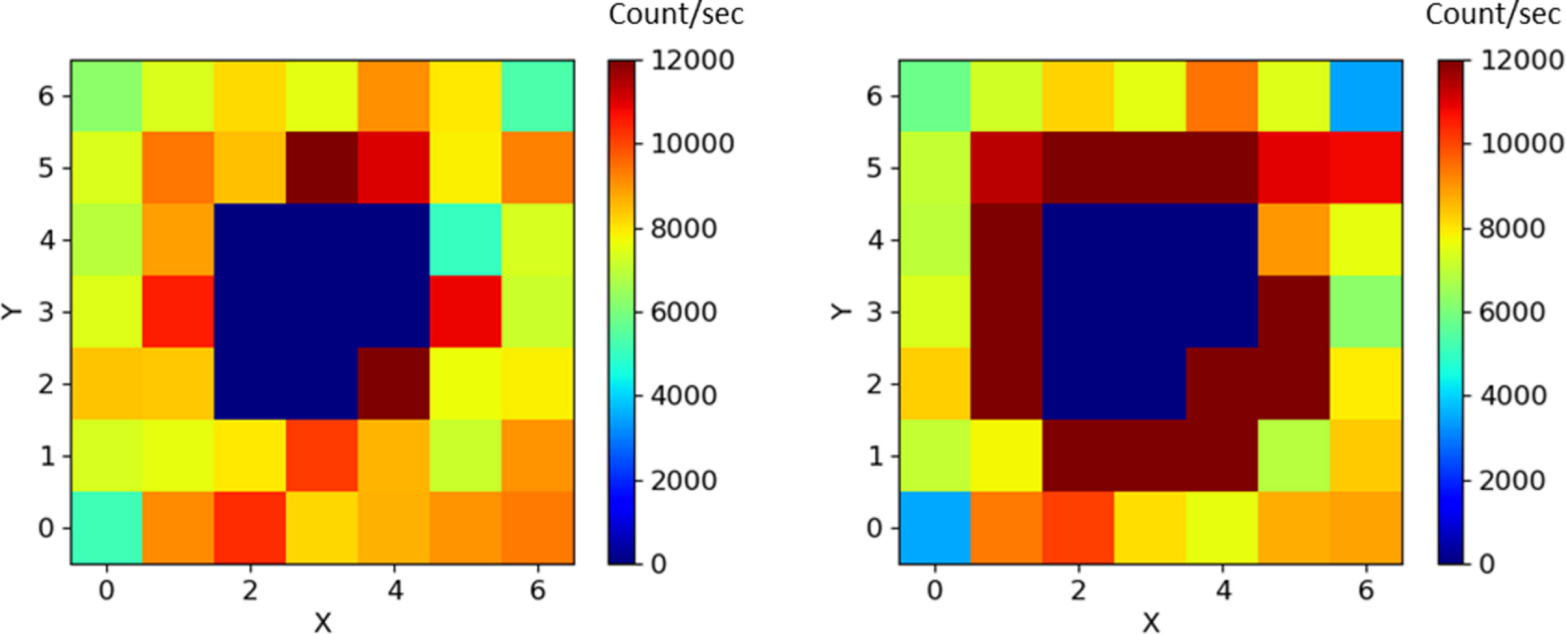
Example of the pixel response around a dead spot under irradiation at 41 keV: initial hit map (left) at the beginning of irradiation and (right) after an irradiation equivalent to 100 Gy.

The detector used for this evaluation is of the hybrid pixel type. Area A contains a dead spot of 8 neighboring pixels that are either not connected or poorly connected to their respective ASICs. These pixels are masked to zero value during experiments. However, during the irradiation measurements in the laboratory, it was observed that the first ring of pixels surrounding this dead spot exhibited unstable behavior. Fig. 11 ▸ presents an example of the hit map near the dead spot at the start of the 41 keV irradiation (left) and after irradiation equivalent to 100 Gy (right). In this measurement, the first ring of pixels surrounding the dead spot exhibits a +25% increase in counts.

**Figure 12:**
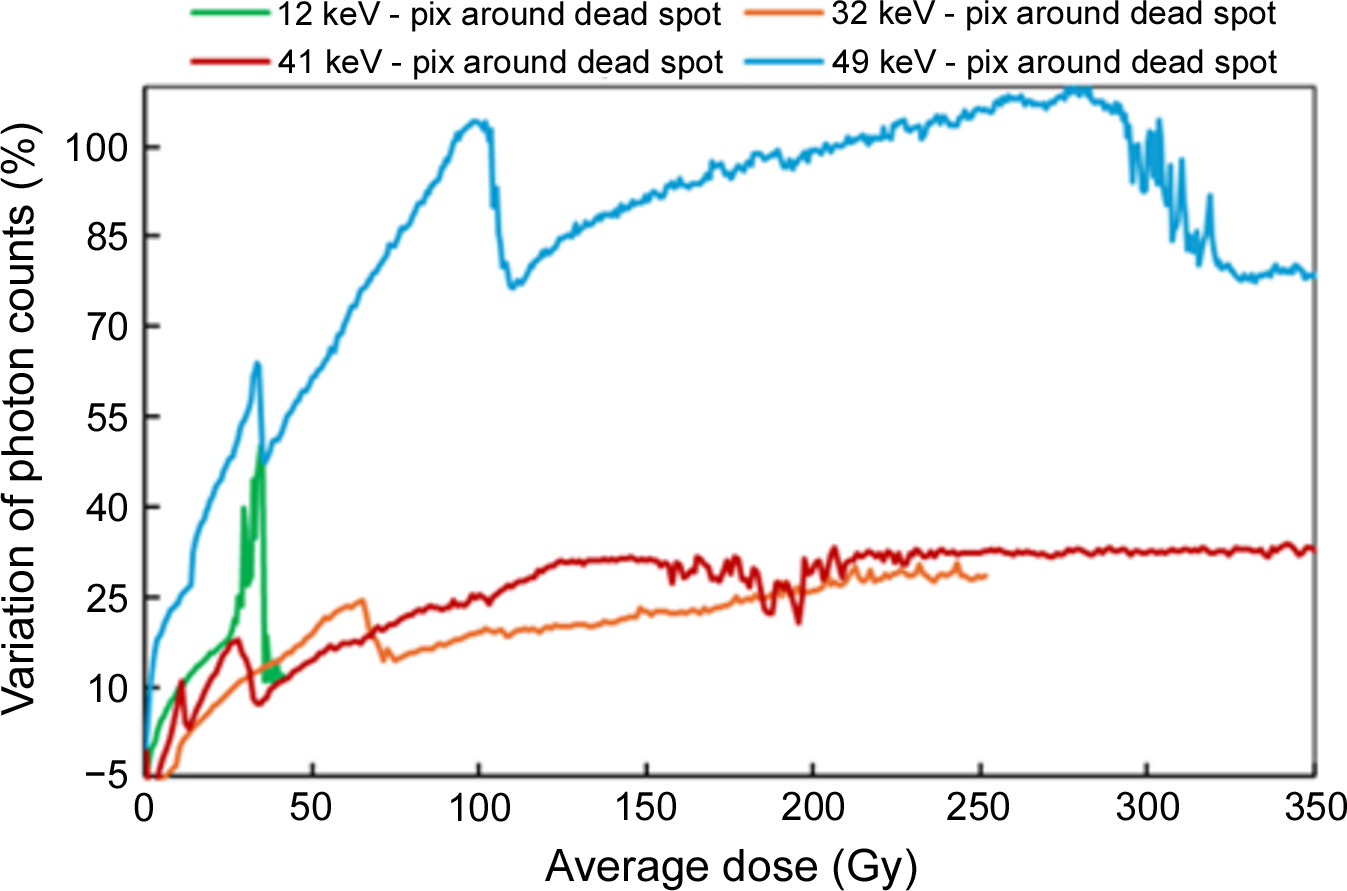
Variation in photon counts of the first ring of pixels surrounding a dead spot as a function of the average dose, at various energies.

Fig. 12 ▸ shows the tracking over time of the pixels surrounding the dead spot at different energies. The instability of these specific pixels is evident, displaying an unpredictable ‘pulsing’ effect. However, since the position of these defects remains fixed over time, it is recommended to update the pixel mask file to include at least one ring of pixels surrounding each ‘dead’ spot.

## 4. Limitations of using CdTe sensors in synchrotron applications

A summary of the CdTe sensor response under irradiation up to 30 Gy, at various energies and dose rates, is provided in Table 2 ▸ for both areas A and B.

As previously mentioned, the variation in performance over time, manifested as a loss of photon counts, is commonly referred to in the literature as a ‘polarization’ phenomenon induced by irradiation (Toyama *et al.*, 2006 ▸).

This phenomenon is most often reported in the case of Schottky contact sensors, where several mechanisms behind the ‘polarization’ phenomenon are explained by ionization of deep acceptors, an increase of the negative space charge due to the holes de-trapping over time, resulting in a strong distortion of the electric field and a shrink of the depletion depth. A decrease in the charge collection efficiency is observed (Okada *et al.*, 2007 ▸; Cola & Farella, 2009 ▸; Nakagawa *et al.*, 2018 ▸), translated into a loss of photon counts in the photon counting detectors.

This irradiation-induced phenomenon also occurs in sensors with ohmic contacts, which operate as photoconductors. Although the underlying mechanism differs from that in Schottky contact sensors (Cola & Farella, 2014 ▸), in ohmic contact sensors it is attributed to a positive charge build-up caused by hole trapping, which affects the electric field. Our study demonstrates that the magnitude of the loss of counts in our detector depends on both the photon energy and the dose rate throughout the sensor.

The polarization effect is observed rather quickly after starting the irradiation, suggesting that the charge trapping appears in the initial tens of minutes. After a period of 30 min to 1 h, once the average dose reaches between 30 Gy and 50 Gy, depending on energy and dose rates, an ‘equilibrium’ is achieved, where the trapping rate of new carriers likely equals the de-trapping rate. This trend is observed in our results, where the photon count curves gradually stabilize over time and dose, following an exponential decay pattern (Figs. 6 ▸ and 7 ▸), consistent with the trapping rate model in semiconductors described by Shockley & Read (1952 ▸).

Our results highlight a correlation between count loss, photon energy and dose rate. As the photon energy increases, photon interactions occur deeper within the CdTe sensor. In this case, the ‘polarization’ effect – gradually weakening the effective electric field due to trapped holes traveling over longer distances – is amplified. A possible explanation would be that the signal amplitude of collected charges decreases over time, resulting in substantial count loss and degradation of the detector response, as shown in Fig. 9 ▸. It would be interesting to study this effect as a function of the front-end electronics settings with another type of detector that allows access to these internal parameters, to check whether it is possible or not to minimize this degradation.

Conversely, at 12 keV, photon interactions occur closer to the front-side surface (the penetration depth is ∼20 µm at this energy). In this case, charge trapping is minimal, and the electric field remains effective, ensuring an efficient charge collection. This results in a slight increase in photon count sensitivity.

This study also highlights two specific cases. First, pixels in defect line areas are more sensitive to irradiation effects (Fig. 10 ▸). While initially corrected by the flat-field file, these defects quickly register significantly fewer counts, potentially causing local artifacts in data analysis that are hard to predict without dedicated beamline experiments. Second, for dead or poorly connected pixels, it is advisable to expand the masked region to include at least one additional ring of surrounding pixels.

Under our photon flux conditions, the CdTe sensor response stabilizes after a certain period. Pre-irradiating the detector could help, but practical constraints – such as sample changes, hutch interventions and limited beam time – make this unrealistic. An alternative approach worth exploring is temperature optimization to accelerate stabilization.

After several hours without voltage, the detector fully recovers without degradation in irradiated areas, indicating that irradiation-induced polarization is reversible under these conditions. However, recovery is slow (at least 3–5 h) and attempts to quickly reset the sensor during or after irradiation have failed to restore the normal count rate. This approach is also impractical for synchrotron experiments requiring continuous data acquisition.

## 5. Summary and conclusions

A laboratory test campaign was conducted on the photon-counting X-ray imaging detector, the LAMBDA 750k, to evaluate the response of its ohmic-type CdTe sensor under 12–49 keV X-ray irradiation. A performance variation over time, commonly referred to as the ‘polarization’ effect induced by irradiation, resulting in a loss of photon counts, was observed and precisely quantified for several dose rates and photon energies. Efforts were made to replicate the realistic conditions of the user’s synchrotron experiment as closely as possible, particularly in terms of photon flux. Despite the detector featuring an ohmic-type contact, which is expected to be less sensitive to the ‘polarization’ effect, an unexpected photon count loss exceeding −4% for energies above 32 keV was observed over time under the conditions described in the previous sections.

The correlation between the loss of photon counts over time as a function of the dose rate and photon energy has been demonstrated in a reproducible manner across two different imaging areas of the detector. Notably, a significant and rapid loss of photon counts of about −11% was observed at 49 keV, even with a very moderate dose rate of 150 mGy min^−1^ (equivalent to 5000 photons s^−1^ pixel^−1^). The measurements conducted in this study provide a quantitative understanding of how sensitive the response of CdTe sensors can be under prolongated X-ray irradiation within this energy range. This information is crucial to consider when conducting experiments at synchrotron facilities, as the polarization effect observed in this study can impact the quality of data and could limit the use of such detectors, depending on the measurement requirements and the experimental conditions in terms of dose.

At several beamlines of SPring-8, maximum photon energies reach up to 115 keV, and we anticipate a significant increase in the number of such high-energy beamlines following the upgrade to SPring-8-II. For this reason, we plan to conduct similar assessments at these higher energies to complement this work in the near future. This study provides reference measurements that can be used to quantitatively compare the responses of high-*Z* sensor materials like CdZnTe, which looks like a promising sensor material for future synchrotron applications.

## Figures and Tables

**Table 1:** Correspondence between photon flux, dose rate, average dose throughout the pixel and the peak dose at the surface, applied to the CdTe sensor in area A, at various photon energies

Photon energy (keV)	Photon flux (photons s^−1^ mm^−2^)	Average dose rate (mGy min^−1^)	Average dose throughout the sensor / peak dose at the surface (Gy)
11.9	6.52 × 10^6^	142	42 / 2010
	4.09 × 10^6^	88	7 / 335
	1.75 × 10^6^	38	7 / 315
	1.10 × 10^6^	24	6 / 310
	4.72 × 10^5^	10	6 / 300
32.2	4.41 × 10^6^	230	253 / 1995
	2.80 × 10^6^	146	40 / 320
	1.20 × 10^6^	62	39 / 305
41.5	3.16 × 10^6^	222	389 / 1990
	2.35 × 10^6^	165	63 / 320
	1.36 × 10^6^	96	60 / 305
49.1	1.61 × 10^6^	129	489 / 1860

**Table 2:** Variation in photon counts in areas A and B as a function of the dose rate and energy for an average dose of 30 Gy

Tested area	Photon count variation at 12 keV	Photon count variation at 32 keV	Photon count variation at 41 keV	Photon count variation at 49 keV
200 mGy min^−1^ ≤ dose rate range ≤ 300 mGy min^−1^				
A	Not possible with the current setup	−4.0 ± 0.4%	−10.8 ± 1.4%	Not possible with the current setup
B		−3.4 ± 0.3%	−7.9 ± 1.2%	

100 mGy min^−1^ ≤ dose rate range ≤ 200 mGy min^−1^				
A	+2.7 ± 0.2%	−3.3 ± 0.3%	−8.3 ± 1.4%	−11.2±1.4%
B	+3.6 ± 0.3%	−2.0 ± 0.2%	−6.1 ± 0.8%	−11.1±1.4%

Dose rate range ≤ 100 mGy min^−1^				
A	+1.5 ± 0.1%	−2.3 ± 0.2%	−7.1 ± 1.2%	–
B	+1.7 ± 0.1%	−1.4 ± 0.2%	−5.2 ± 0.6%	–
